# Regulation of Tau Protein on the Antidepressant Effects of Ketamine in the Chronic Unpredictable Mild Stress Model

**DOI:** 10.3389/fpsyt.2019.00287

**Published:** 2019-04-30

**Authors:** Gehua Wen, Hui Yao, Yanning Li, Runtao Ding, Xinghua Ren, Yaqing Tan, Weishu Ren, Hao Yu, Xiaoni Zhan, Xiaolong Wang, Enyu Xu, Jun Yao, Guohua Zhang, Yan Lu, Xu Wu

**Affiliations:** ^1^School of Forensic Medicine, China Medical University, Shenyang, China; ^2^Department of Forensic Medicine, School of Basic Medicine, Gannan Medical University, Ganzhou, China; ^3^Key Laboratory of Health Ministry in Congenital Malformation, the Affiliated Sheng Jing Hospital of China Medical University, Shenyang, China

**Keywords:** depression, hippocampus, Tau protein phosphorylation, synapse, ketamine

## Abstract

Tau protein is known to play an important role in maintaining microtubule assembly and stabilization, and maintaining the normal morphology of neurons, but several studies have found that chronic stress leads to Tau hyperphosphorylation. A large number of clinical trials have found that ketamine, which is an N-methyl-D-aspartate receptor antagonist, produces a rapid, long-lasting, and potent antidepressant effect in patients suffering from major depression. This rapid antidepressant effect of ketamine, which involves many mechanisms, has attracted wide attention. However, the relationship between ketamine’s antidepressant effects and Tau protein has rarely been examined. We used C57BL/6 and Tau KO mice exposed to 42 days of chronic unpredictable mild stress (the CUMS model) to investigate the effect of ketamine on behavioral changes and synaptic functioning of the hippocampus. The results showed that a single treatment of ketamine rapidly relieved the CUMS-induced anhedonia, depression-like, and anxious behaviors of the C57BL/6 mice. The abnormal behaviors were accompanied by increased levels of specific alterations of hyperphosphorylated Tau protein in cytoplasm and synapse in the hippocampus of the C57BL/6 mice, but ketamine reduced the aggregation of hyperphosphorylated Tau protein only in the synapse. We also found that CUMS exposure reduced the levels of GluA1 and PSD95 in the hippocampus of the C57BL/6 mice and that these deficits were reversed by ketamine. However, the Tau KO mice did not develop any stress-induced depressive behaviors or deficits of hippocampal function. The antidepressant effect of ketamine may decrease the levels of hyperphosphorylated Tau protein in synapse of C57BL/6 mice.

## Introduction

Depressive disorders were reported to be the leading cause of the global disease burden in 2014, with prevalence rates throughout the world ranging from 3% to 17% (1). Studies indicate that early-life depression is associated with a 2–4-fold increased risk of developing Alzheimer’s disease (AD) (2, 3), the main feature of which is the aggregation of neurofibrillary tangles (NFTs) after Tau hyperphosphorylation, which is one of the main pathological characteristics of AD (4). The Tau protein, one of the microtubule-associated proteins (MAPs), plays an important role in maintaining neuron morphology and promoting axonal development in the cytoskeleton system. Tau proteins are the transport channels of axons and dendrites (5), and phosphorylated Tau regulates this function during normal neuron maturation. However, Tau can evoke neurotoxicity when it is abnormally hyperphosphorylated (6). Hyperphosphorylation of Tau protein affects intracellular material transportation and eventually leads to hippocampus neuron atrophy, which is also a key factor in the pathogenesis of AD (7).

Recent studies on C57BL/6 mice have shown that chronic unpredictable mild stress (CUMS) can induce hyperphosphorylation of Tau protein at the pSer202, pThr231, pSer262, and pSer396/404 sites of hippocampal neurons (8). Yang et al. (9), who produced hyperphosphorylation of Tau at sites Ser356 and Thr231 in the hippocampus and prefrontal cortex of C57BL/6 mice by CUMS, found that fluoxetine reduced the levels of hyperphosphorylated Tau protein of whole protein after 3 weeks of treatment. Although fluoxetine is widely used, it often takes weeks to reach efficacy. Ketamine, an ionotropic glutamatergic NMDA receptor antagonist, has been used by millions of people as an anesthetic over the past 40 years (10). In recent years, clinical studies have found that ketamine produced an antidepressant effect within 2–4 h that lasts from several days to 2 weeks. It has also been found to reduce the suffering of patients with major depressive disorder (11–13), but its mechanism of action is not clear. Therefore, the rapid and effective antidepressant effects of ketamine have become a “hot spot” in the field.

Studies have found that the expression of the synaptic proteins GluR1 and PSD95 on the hippocampal neuron membrane decreased after rats showed depression-like behavior (14). GluR1, a major subunit of AMPA, plays a vital role in maintaining synaptic plasticity and the processing of long-term depression (LTD) and long-term potentiation (LTP) (15). AMPA receptors are transported to the postsynaptic membrane and then anchored on the PSD95 scaffold protein of the postsynaptic membrane to perform the function of synaptic transmission (16). PSD95 is the most important and abundant scaffold protein on the postsynaptic membrane, which mainly exists in mature excitatory glutamate synapses (17). PSD95 is involved in the formation and reconstruction of synapses, and decreases in its expression indicate the loss of synapses (18). The antidepressant effect of ketamine is related to the rapid synthesis of brain-derived neurotrophic factors (BDNFs) (19), m-TOR (rapamycin target protein) (20), and eukaryotic elongation factor 2 (eEF2) (21). Therefore, it is crucial to explore the role of Tau protein in the pathogenesis of depression and whether Tau protein is involved in the ketamine antidepressant process.

## Materials and Methods

### Animals

The study’s subjects were 63 adult male C57BL/6 mice and 63 adult male Tau knockout (KO) mice. The C57BL/6 mice were 2 months old (20–23 g) from the Laboratory Animal Center of China Medical University, and the 63 Tau KO mice [B6.129S4-Mapttm/(EGFP)Kit] were 2 months old (20–23 g) from the Jackson Laboratory, bred at the China Medical University. The mice were housed individually in ventilated cages on a 12-h light/dark cycle with free access to water and food. Each strain of mice was randomly divided into three groups (n = 21 per group): a control (CON) group, and two experimental groups—a CUMS group and a CUMS plus ketamine treatment (CUMS+KET) group. The experimental animals were exposed to a series of mild unpredictable stressors for 42 days, as explained below. CUMS+KET and CUMS groups were given an intraperitoneal injection of ketamine hydrochloride 10 mg/kg (14, 22) and the identical dose of normal saline at 8:00 am on the 43rd day, respectively. The sucrose-preference test (SPT) and the open-field test (OFT) were conducted on the 44th day, the elevated plus-maze (EPM) test was conducted on the 45th day, and the forced-swim test (FST) was conducted on the 46th day. All animal procedures were conducted in accordance with the Guidelines for the Care and Use of Laboratory Animals of China Medical University. The protocols were approved by the Institutional Animal Care and Use Committee of China Medical University.

### Chronic Mild Unpredictable Stress

The mice were exposed to a variable sequence of unpredictable and mild stressors (23–28) for 6 weeks. These stressors included 45° cage tilting for 4 h, damp sawdust (200 ml water, 200 g sawdust in a cage) for 4 h, 24 h of food or water deprivation, restriction in a 115 mm × 29 mm cylindrical plastic restrainer for 4 h, alterations of the light–dark cycle, 120-db noise overnight, and strobe lights overnight. The animals were exposed to two stressors everyday; stressors were never presented simultaneously.

### Ketamine Administration

Ketamine hydrochloride (Fujian Gutian Pharmaceutical Co., Ltd., Gutian, Fujian, China) was dissolved in physiological saline immediately before use. An intraperitoneal injection of ketamine (10 mg/kg) or the identical dosage of saline was administered to the mice on the 43rd day.

### Behavioral Tests

#### Sucrose-Preference Test

We used the SPT to assess anhedonia, which is the core symptom of depression. The mice were given two bottles containing 2% sugar water (Tianjin Guangfu Technology Development Co. Ltd). on the first day, and a bottle of water and a bottle of 2% sucrose on the second day. On the third day, all the mice were deprived of water for 24 h, and on the fourth day, they were given a bottle of 2% sucrose solution and a bottle of water for 1 h at 8:00–9:00 am. The procedure was originally described by Willner et al. (29). Sucrose and water consumption were determined by measuring the change in the volume of the fluid consumed; the sucrose-preference percentage = sugar water consumption/(sugar water consumption + water consumption) × 100%.

#### Open-Field Test

The OFT used a procedure described in a previous report (30). Each mouse was tested separately in an open field to assess their locomotor activity and “anxious” behaviors. The open field consisted of a plastic structure of 40 cm × 40 cm × 30 cm, with a white floor. We defined the area adjacent to the surrounding wall as the peripheral area and the rest of the field as the central area (23 cm × 23 cm). The observation of the animals and the data analysis were performed using the SMART^™^ tracking software system (San Diego Instruments, San Diego, CA). The total distance traversed in the open field and time spent in the central area were observed for 10 min.

#### Elevated Plus-Maze

The EPM used a procedure described in a previous report (31) to evaluate the “anxiety” of the mice. Each mouse was placed in the EPM, which consisted of two open arms (30 cm × 8 cm) and two closed arms (30 cm × 8 cm × 16 cm) that were elevated 50 cm above the floor in a dimly illuminated room. The open arms and closed arms were perpendicular to each other, and cameras were installed directly above the maze. Each mouse was placed on the central platform of the maze, with its head aligned to one of the open arms. The mouse freely explored the maze for 5 min. The time spent in the open arms was recorded by the SMART^™^ tracking analysis system.

#### Forced-Swim Test

The FST was used to evaluate the “depression-like” behavior of the mice (32, 33). Each mouse was placed into a transparent cylinder (10 cm in diameter, depth of 22 cm), filled with water (23ºC to 25ºC). The mice could not touch the bottom for support. The test was conducted for 6 min: the first 2 min was an adaption phase, after which the immobility of the mouse in the water was recorded for 4 min (immobility refers to the absence of active struggle, with the mouse’s body floating in the water).

### Western Blot

The mice were anesthetized with excessive sevoflurane and decapitated at the end of the last test. The brain was quickly separated from the skull, and the hippocampus was dissected from both hemispheres, while the brain was on an ice plate. The hippocampus samples were kept at −80ºC until use.

The analysis of the synaptosomal fraction was performed using the Syn-PER Reagent (Thermo Scientific) with a protease/phosphatase (50×) inhibitor (P1046, Beyotime, Shanghai, China). Briefly, samples were centrifuged at 1,200×*g* for 10 min, and the remaining supernatant was centrifuged at 15,000×*g* for 20 min to obtain synaptosome pellets and supernatant cytoplasm. In order to verify whether Tau KO mice express Tau protein, we extracted the whole protein of C57BL/6 mice and Tau KO mice, and samples were dissolved with RIPA (P0013B, Beyotime, Shanghai, China) and then centrifuged at 1,200×*g* for 5 min. Then, protein concentrations were determined using a bicinchoninic acid (BCA) Protein Assay Kit (P0012, Beyotime, Shanghai, China). Equal amounts of protein (10 μl) for each Western-blot sample were loaded into 12% sodium dodecyl sulfate polyacrylamide gel electrophoresis (SDS-PAGE) and then transferred to polyvinylidence difluoride (PVDF) membranes (Millipore). After being blocked with 8% nonfat milk in Tris-buffered saline–Tween-20 (TBST-20) at room temperature for 2 h, the membranes were incubated with primary antibodies [total Tau, 1:2,000, BD Biosciences; phospho-Tau pSer199, 1:2,000, Abcam; phospho-Tau AT8 (pSer202+Thr205), 1:2,000, Invitrogen; phospho-Tau pSer396, 1:1,000, Life Technologies; phospho-Tau pSer404, 1:2,000, Abcam; GluR1, 1:2,000, Millipore; PSD95, 1:2,000, Abcam; β-actin, 1:5,000, ZSGB-BIO] overnight at 4°C. The blots were washed three times in TBST the next day and incubated with appropriate horseradish peroxidase-conjugated secondary antibodies at room temperature for 1.5 h. The blots were visualized with a luminal reagent, and immunoreactive bands were imaged by Electrophoresis Gel Imaging Analysis System (Tanon 5500, Shanghai, China). Quantification of proteins was performed using National Institutes of Health (NIH) ImageJ software and then normalized to the respective actin band.

### Statistical Analysis

One-way analysis of variance (ANOVA) and Tukey *post hoc* tests were used to compare differences between the groups. T test was used to compare C57BL/6 mice and Tau KO mice of their respective CON groups. The statistical analyses were conducted with GraphPad Prism 6.0 software; *p*-values less than 0.05 were considered statistically significant.

## Results

**Figure 1:**
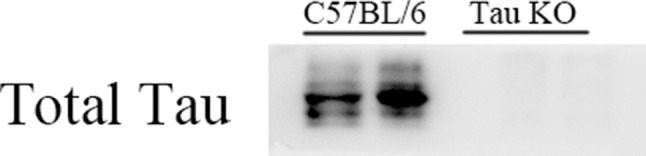
Tau knockout (KO) mice did not express Tau protein.

In this study, C57BL/6 and Tau KO mice were used. For Tau KO mice, Western blot results of anti-Tau protein specific antibodies showed that Tau KO mice had no positive bands at 50–55 kDa (Tau protein position), while C57BL/6 mice normally expressed Tau protein (**Figure 1**).

The C57BL/6 mice exhibited depressive behaviors, including anxiety, anhedonia, and depression-like after CUMS, and ketamine treatment alleviated their depressive behavior. Tau KO mice did not exhibit depressive behavior after CUMS. After treatment with ketamine at the same dose, there was no significant change in the behavioral results between CUMS+KET and CON and CUMS groups.

### Sucrose-Preference Test

**Figure 2:**
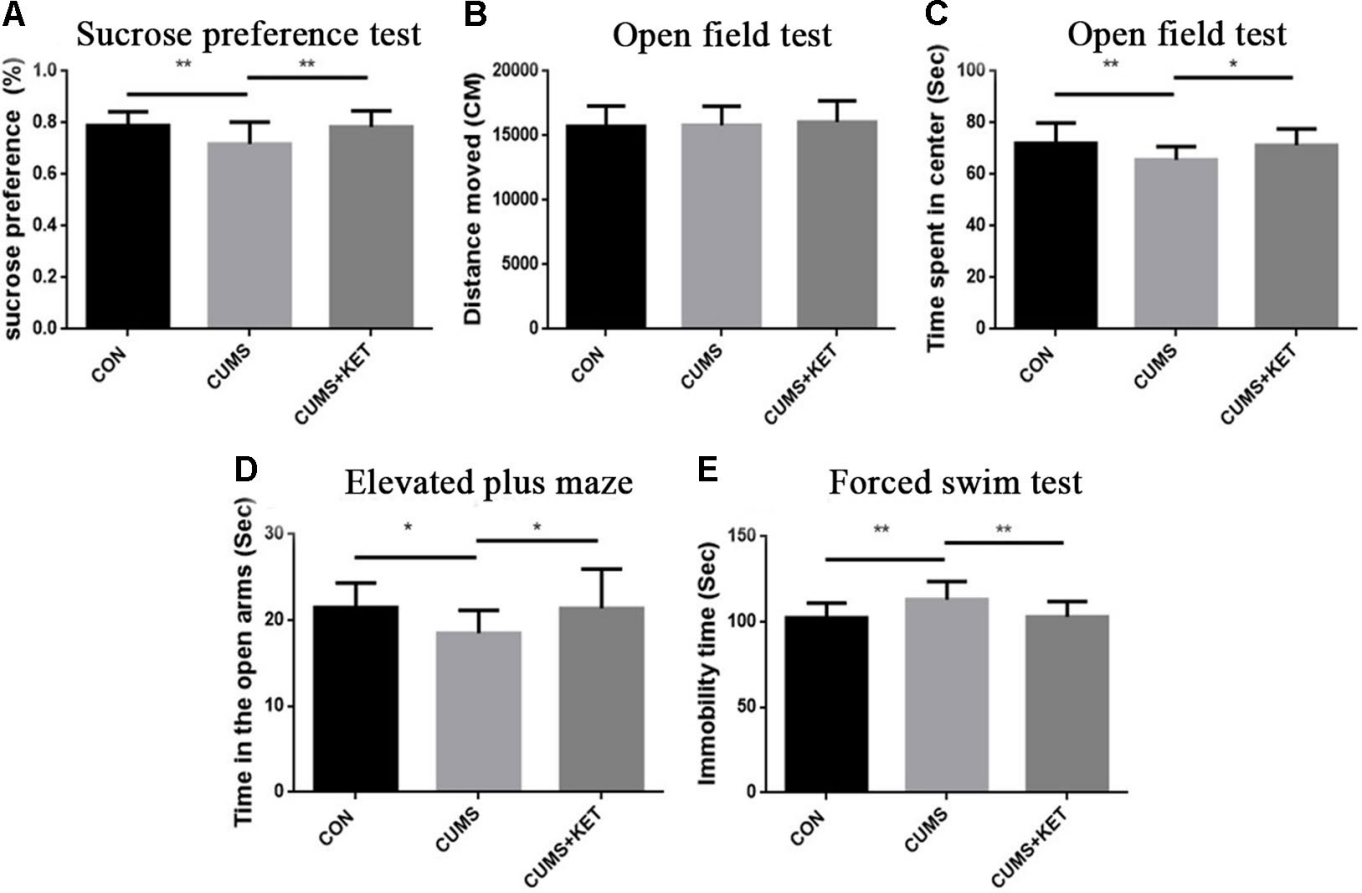
Effects of chronic unpredictable mild stress (CUMS) on the anhedonia, anxiety, and depression-like behaviors of C57BL/6 mice, and ketamine reversal of these behaviors. **(A)** Preference for the sucrose solution on the sucrose-preference test. **(B)** Total distance traversed in the open-field test. **(C)** Time spent in the center area of the open-field test. **(D)** Time in the open arms of the elevated plus-maze. **(E)** Duration of immobility in the forced-swim test. Data are expressed as mean ± SD; n = 21 per group. One-way ANOVA was used to compare differences among the CON, CUMS, and CUMS+KET groups (*p < 0.05, **p < 0.01).

**Figure 3:**
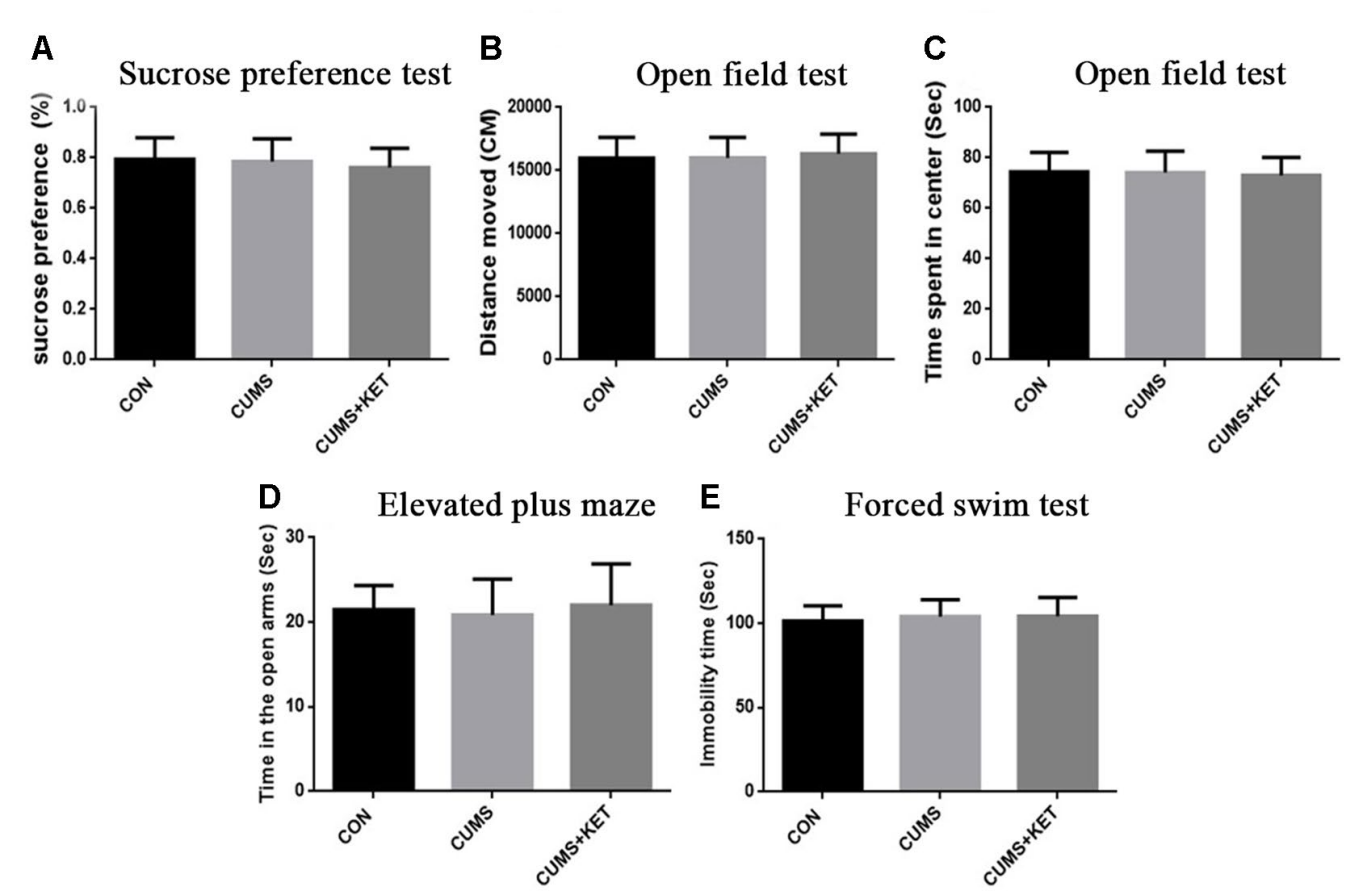
Effects of CUMS on the anhedonia, anxiety, and depression-like behaviors for Tau KO mice. **(A)** Preference for the sucrose solution on the sucrose-preference test. **(B)** Total distance traversed in the open-field test. **(C)** Time spent in the center area of the open-field test. **(D)** Time in the open arms of the elevated plus-maze. **(E)** Duration of immobility in the forced-swim test. Data are expressed as mean ± SD; n = 21 per group. One-way ANOVA was used to compare difference among the CON, CUMS, and CUMS+KET groups.

The CUMS procedures simulated anhedonia in the C57BL/6 mice, and ketamine reduced this behavior. **Figure 2A** shows the sucrose preference of the three groups of C57BL/6 mice. Six weeks of chronic stress produced a significant decrease in sucrose consumption in the CUMS group compared to the CON group of C57BL/6 mice, and ketamine reversed this decrease [**Figure 2A**: *F*_(2,60)_ = 7.222, *p* < 0.05]. However, there was no significant difference in sucrose preference between the CUMS and CON groups of Tau KO mice, and the CUMS+KET group did not differ significantly for the CON and CUMS groups of Tau KO mice (**Figure 3A**: *F* = 0.8971, *p* > 0.05).

### Open-Field Test

There was no significant difference in the total distance traversed by the CON, CUMS, and CUMS+KET groups of C57BL/6 mice [**Figure 2B**: *F*_(2,60)_ = 0.2402, *p* > 0.05] or by the CON, CUMS, and CUMS+KET groups of Tau KO mice [**Figure 3B**: *F*_(2,60)_ = 0.3049, *p* > 0.05]. However, the CUMS group of C57BL/6 mice spent significantly more time in the central area than did the CON group. Treatment with ketamine decreased time in the center area, indicating that the anxiety behavior of the C57BL/6 mice was alleviated by ketamine treatment [**Figure 2C**: *F*_(2,60)_ = 5.838, *p* < 0.05]. There was no significant difference in spent time in central area among the three groups of Tau KO mice [**Figure 3C**: *F*_(2,60)_ = 0.1705, *p* > 0.05].

### Elevated Plus-Maze

CUMS significantly reduced the duration of time spent in the open arms of the maze by C57BL/6 mice, and antidepressant treatment with ketamine reversed this measure of anxious behavior [**Figure 2D**: *F*_(2,60)_ = 5.936, *p* < 0.05]. There was no significant difference in time spent in the open arms between the three groups of Tau KO mice after stress exposure or ketamine treatment [**Figure 3D**: *F*_(2,60)_ = 0.4545, *p* > 0.05].

### Forced-Swim Test

**Figure 2E** shows that the time the CUMS group was immobile during the FST was significantly longer than that of the CON group, indicating greater depression-like after CUMS exposure among the C57BL/6 mice; however, ketamine treatment significantly reduced the duration of immobility [*F*_(2,60)_ = 8.656, *p* < 0.05]. The duration of immobility in the FST did not differ significantly among the three groups of Tau KO mice [**Figure 3E**: *F*_(2,60)_ = 0.4808, *p* > 0.05].

C57BL/6 mice showed depressive behavior after CUMS, whereas Tau KO mice did not show depressive behavior after CUMS. We used *t*-tests to compare the C57BL/6 mice and Tau KO mice of their respective CON groups. The results showed that there was no statistical difference in the behavioral manifestations of C57BL/6 and Tau KO mice not exposed to CUMS: sucrose preference, *t* = 0.82, *p* > 0.05; total distance of open field test, *t* = 0.62, *p* > 0.05; time spent in the center of the open field, *t* = 0.33, *p* > 0.05; EPM, *t* = 0.99, *p* > 0.05; FST, *t* = 0.75, *p* > 0.05.

CUMS-induced total Tau and phosphorylation of Tau at pSer396 and pSer404 increased in the cytoplasm and synapses, while the expression level of phosphorylated Tau at pSer199 and AT8 sites remained unchanged. Ketamine decreased hyperphosphorylated Tau protein in synapses at the pSer396 and pSer404 sites.

**Figure 4:**
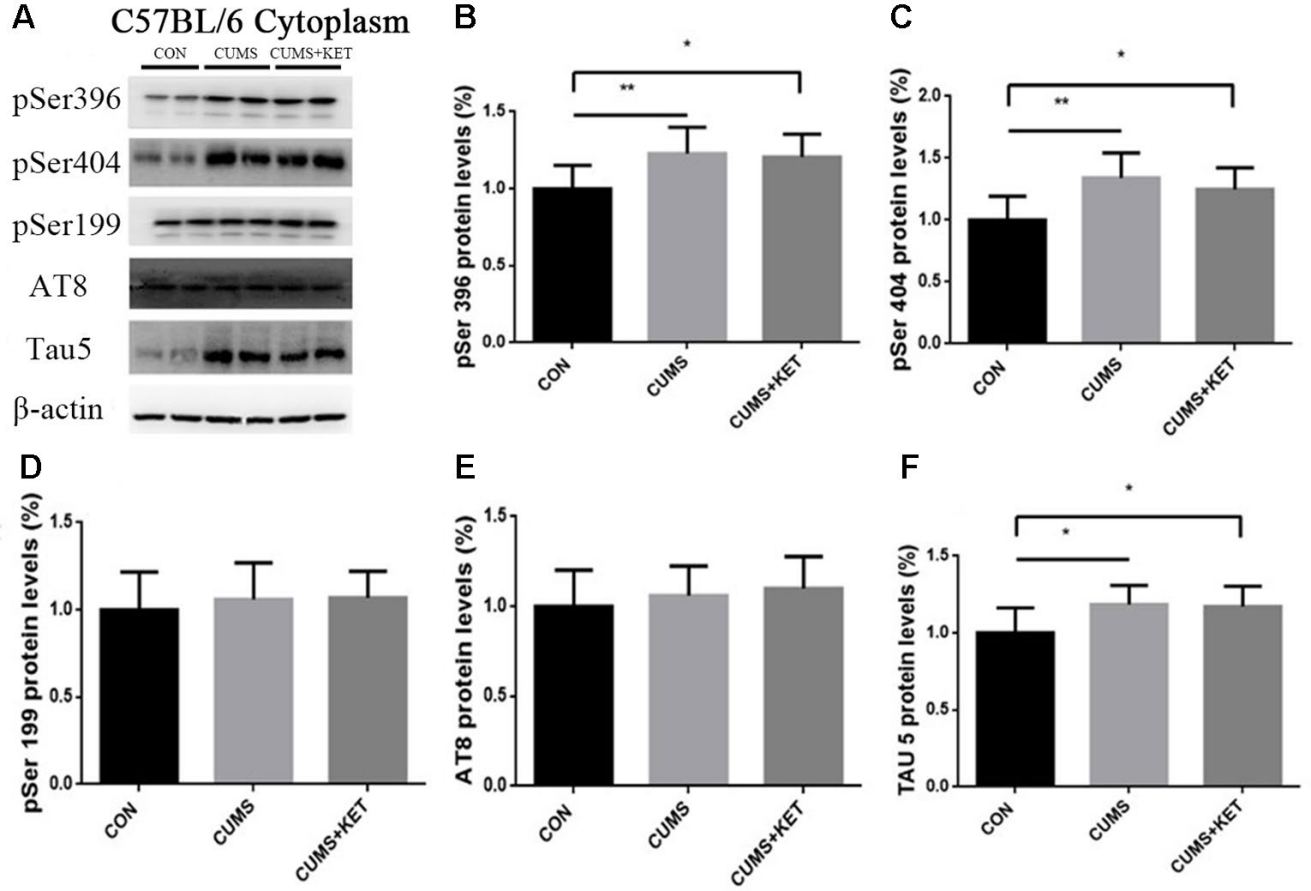
The effects of CUMS and ketamine on Tau protein in the cytoplasm of the hippocampus of C57BL/6 mice. **(A)** Total Tau and hyperphosphorylated Tau protein in the cytoplasm of the hippocampus. **(B,** **C,** and **F)** After CUMS, the levels of pSer396, pSer404, and total Tau protein increased; ketamine did not change cytoplasm levels. **(D** and **E)** Levels of pSer199 and AT8 of Tau protein did not change after CUMS or ketamine treatment. Data are expressed as mean ± SD; n = 10 per group. One-way ANOVA was used to compare differences among the CON, CUMS, and CUMS+KET groups.

**Figure 5:**
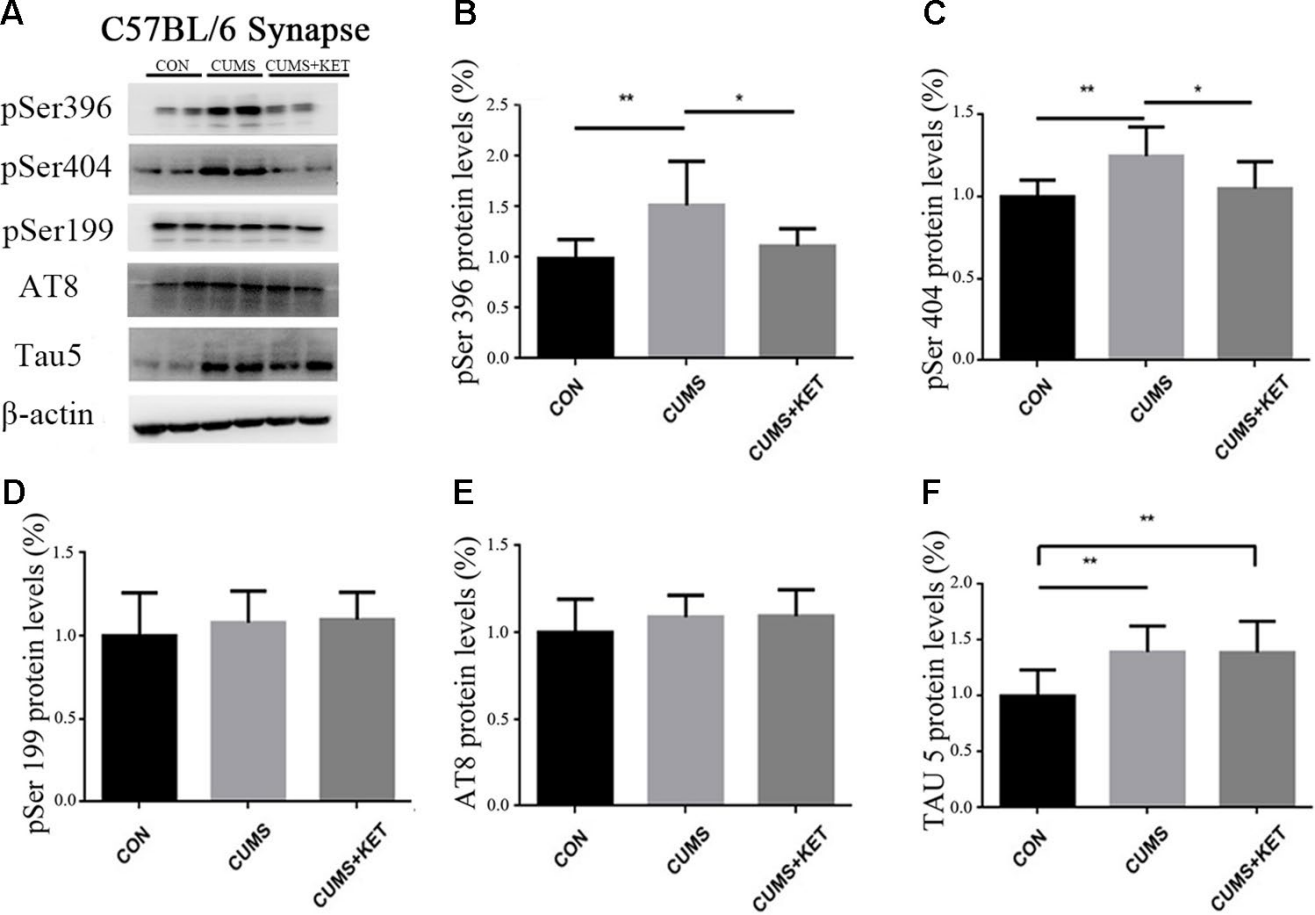
The effects of CUMS and ketamine on Tau protein in the synapses of the hippocampus of C57BL/6 mice. **(A)** Total Tau and hyperphosphorylated Tau protein in the synapses of the hippocampus. **(B,** **C,** and **F)** After CUMS, the levels of pSer396, pSer404, and total Tau protein increased; ketamine decreased the levels of hyperphosphorylation at pSer396 and pSer404 sites in the synapses, but not total Tau. **(D** and **E)** Levels of pSer199 and AT8 did not change after CUMS and ketamine treatment did not alter the levels in the synapses. Data are expressed as mean ± SD; n = 10 per group. One-way ANOVA was used to compare differences among the groups (*p < 0.05, **p < 0.01).

We observed the effect of CUMS on Tau and its phosphorylation state in the cytoplasm and synapses of the hippocampus of C57BL/6 mice in **Figure 4A** **and** **Figure 5****A**. CUMS induced a significant increase in cytoplasm levels of total Tau, accompanied by increased levels at pSer396 and pSer404, as seen in **Figure 4F** [*F*_(2,27)_ = 5.304, *p* < 0.05], **Figure 4B** [*F*_(2,27)_ = 6.370, *p* < 0.05], and **Figure 4C** [*F*_(2,27)_ = 8.550, *p* < 0.05]. However, there was no change after CUMS at pSer199 [**Figure 4D**: *F*_(2,27)_ = 0.3675, *p* > 0.05] or AT8 [**Figure 4E**: *F*_(2,27)_ = 0.7435, *p* > 0.05]. We found that CUMS increased total Tau levels [**Figure 5F**: *F*_(2,27)_ = 8.088, *p* < 0.05], as well as the levels of hyperphosphorylation at pSer396 [**Figure 5B**: *F*_(2,27)_ = 8.963, *p* < 0.05] and pSer404 [**Figure 5C**: *F*_(2,27)_ = 7.374, *p* < 0.05] in the synapses of the hippocampus of C57BL/6 mice, but not the pSer199 [**Figure 5D**: *F*_(2,27)_ = 0.6130,**** *p* > 0.05] and AT8 sites [**Figure 5E**: *F*_(2,27)_ = 1.100, *p* > 0.05]. Total and hyperphosphorylated Tau protein did not change in the cytoplasm after ketamine treatment. However, at the synapse, ketamine decreased the levels of hyperphosphorylation at the pSer396 (**Figure 5B**: *p* < 0.05) and pSer404 sites (**Figure 5C**: *p* < 0.05). Total Tau did not change at the synapse after ketamine treatment (**Figure 5F**).

### Ketamine Reversed the CUMS-Induced Decrease in GluA1 and PSD95 in the Hippocampus Synapses of C57BL/6 Mice

**Figure 6:**
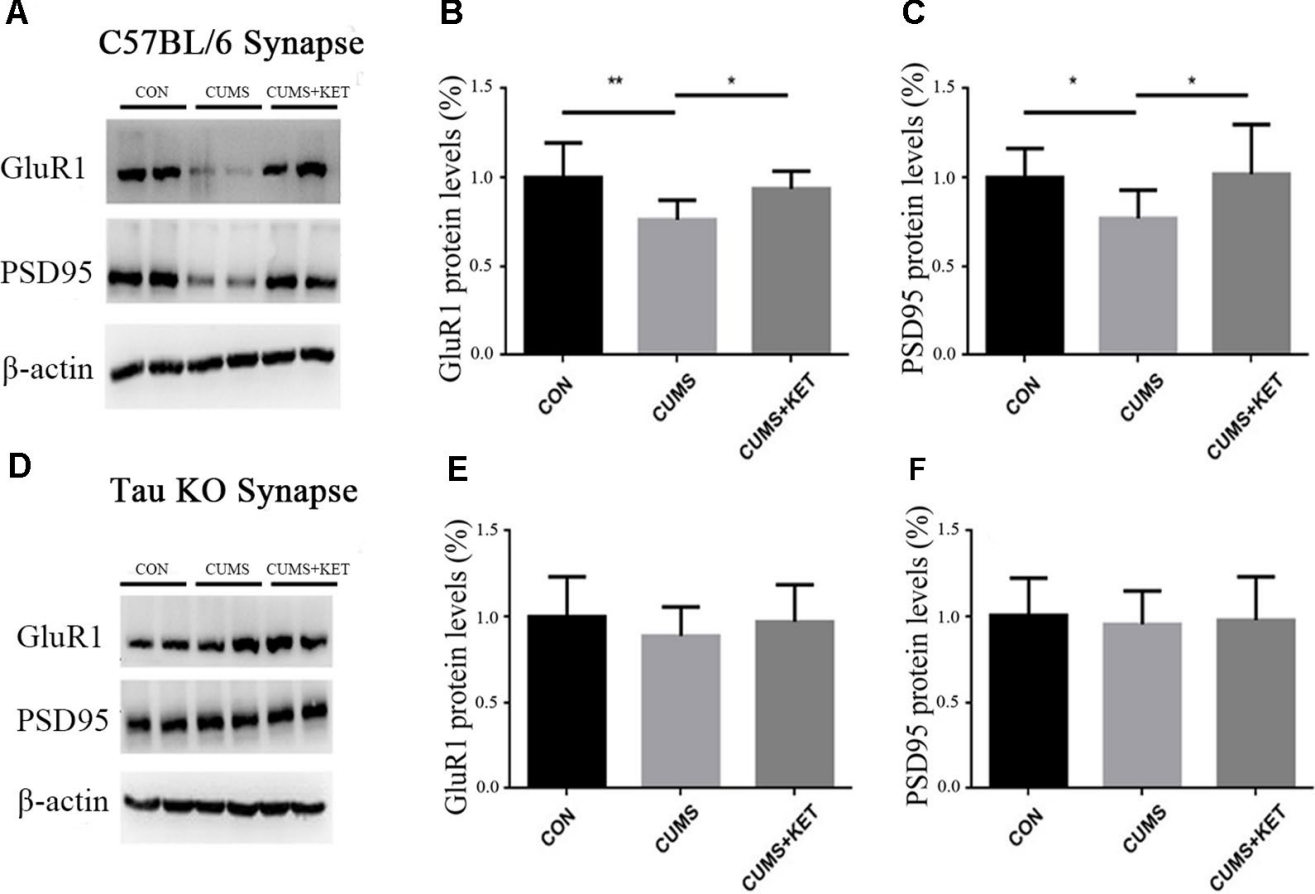
CUMS decreased the levels GluA1 and PSD95 of hippocampus synapses in C57BL/6 mice, with a rapid reversal by ketamine. CUMS did not decrease the levels of GluA1 and PSD95 of hippocampus synapses of Tau KO mice, and ketamine had no effect on the Tau KO mice. **(A)** Representative Western blot images of the GluA1 and PSD95 of C57BL/6 mice are shown. **(B** and **C)** CUMS decreased the expression of GluA1 and PSD95, and this deficit was reversed by a single dose of ketamine. **(D)** Representative Western blot images of GluA1 and PSD95 of Tau KO mice are shown. **(E** and **F)** CUMS and ketamine did not change the levels of GluA1 and PSD95 of Tau KO mice. Results were quantified and expressed as the mean ± SD percent of the control; n = 10 (*p < 0.05, **p < 0.01).

CUMS exposure in our experiment decreased the levels of GluA1 and PSD95 in the hippocampus synapses of C57BL/6 mice (**Figure 6A**). A single injection of ketamine treatment reversed the deficits of these synaptic proteins [**Figure 6B**: *F*_(2,27)_ = 7.554, *p* < 0.05; **Figure 6C**: *F*_(2,27)_ = 4.495, *p* < 0.05].

CUMS in our experiment did not change the levels of GluA1 and PSD95, and ketamine treatment had no effects on the Tau KO mice (**Figure 6D**) [**Figure 6E**: *F*_(2,27)_ = 0.7851, *p* > 0.05, **Figure 6F**: *F*_(2,27)_ = 0.8668, *p* > 0.05].

## Discussion

A large number of animal experiments are needed to study the etiology of depression and test antidepressants in addition to the study of clinical patients with depression. Therefore, it is particularly valuable to establish effective animal models. Common animal models of depression include the CUMS model (25), social defeat model (34), chronic restraint stress model (35), maternal separation model (36), and the repeated corticosterone injection model (37). We chose to use the CUMS model because CUMS is the best model to mimic the long-term, low-intensity stress of human daily life.

We used CUMS to establish a depression model in C57BL/6 mice followed by ketamine treatment and conducted behavioral tests on three groups of mice. There was no significant group difference in the total distance traversed in the OFT, indicating that the locomotor activity of mice in each group was not affected. The CUMS group, which was exposed to stress, spent increased time in the center area of the OFT and decreased time in the open arms of the EPM, indicating the C57BL/6 mice were anxious, and their decrease in sucrose preference indicated that these mice exhibited anhedonia. The prolonged duration of immobility time in the FST indicated that the mice experienced desperation. The CUMS group showed the core symptoms of depression—anhedonia and depression-like behaviors, indicating that our depression model was successfully established. Compared with the CUMS group, the CUMS+KET group spent decreased time in the center of the open field, increased time in the open arms of the EPM, increased sucrose preference in the SPT, and significantly less immobility in the FST, indicating that ketamine effectively alleviated the depressive symptoms induced by CUMS.

We used Tau KO mice of the same age to investigate the role of Tau protein in the pathogenesis of depression after undergoing exposure to CUMS exposure and ketamine treatment. Their distance traversed and time in the center area of the OFT, sucrose preference in the SPT, time in the open arms of the EPM, and duration of immobility in the FST did not differ statistically among the three groups (CON, CUMS, and CUMS+KET), showing that the Tau KO mice did not have anhedonia, anxiety, or depression-like behaviors after CUMS, and ketamine did not affect their behaviors. These results suggest that Tau protein plays an important role in the pathogenesis of depression. That is, after Tau protein was knocked out, the mice “escaped” the harmful effects caused by CUMS.

Tau is regulated by its different isomer forms and phosphorylation states. Hyperphosphorylation of Tau protein is generally a key step leading to its migration from axons to dendrites (38). Hyperphosphorylation of Tau protein aggregates in the synapse, leading to synapse loss and impaired neuron function (39, 40). The synapse is the connection between neurons and the key sites of information transmission. Therefore, the hyperphosphorylated Tau protein aggregates in synapses, which is significant for neuron damage. In current studies on depression, hyperphosphorylation forms of Tau protein in the hippocampus are mostly detected by whole protein components (9, 41), but not specifically at the synapse. Therefore, we isolated the cytoplasm and synapses from hippocampal tissue to observe the aggregation of hyperphosphorylated Tau protein in the cytoplasm and synapses, to investigate whether ketamine can affect hyperphosphorylated Tau protein in synapses. We found that C57BL/6 mice showed depressive behavior after CUMS exposure, while the levels of total Tau and hyperphosphorylated Tau at pSer396 and pSer404 increased in the cytoplasm and synapse of hippocampal neurons. After ketamine treatment, hyperphosphorylated Tau decreased at pSer396 and pSer404 sites in the synapses of hippocampal neurons in C57BL/6 mice. These results suggest that ketamine may play an antidepressant role by reducing hyperphosphorylated Tau in the synapse.

Regan found that when Tau protein was hyperphosphorylated at Ser396 in hippocampal slices of Wistar rats, it could induce LTD (42). The pathogenesis of depression is similar to LTD, resulting in decreased synaptic efficiency, synaptic contraction, and loss of function (43). The 3xTg-AD transgenic mouse model is a good model to simulate AD. Studies have found that Tau protein in the hippocampal total protein of 3xTg-AD transgenic mice is over-phosphorylated at Ser396 and Ser262, which leads to decreased expression of synaptic GluR1 and PSD95 (44). We found the expression of GluR1 and PSD95 in hippocampal synapses of C57BL/6 mice was decreased after CUMS, whereas the expression of GluR1 and PSD95 in Tau KO mice was not affected by CUMS. We hypothesized that CUMS decreased the expression levels of GluR1 and PSD95 protein in the hippocampal synapses of C57BL/6 mice, which may be caused by hyperphosphorylated Tau protein expression in hippocampal synapses. Ketamine treatment reduced the expression of hyperphosphorylated Tau protein in the hippocampal synapses of C57BL/6 mice and increased the expression of GluR1 and PSD95. Tau protein may play an important role in both depression disorder induced by CUMS and hippocampal synaptic protein defects, as well as processes in which ketamine plays an antidepressant role.

Tau protein hyperphosphorylation involves many sites; in this experiment, the levels of Tau protein and the phosphorylated Tau protein at sites pSer396 and pSer404 increased in the hippocampal neuronal cytoplasm and synapses of C57BL/6 mice after CUMS. The levels of phosphorylated Tau protein at sites pSer199 and AT8 did not change in the cytoplasm or synapses. We speculate that CUMS may induce Tau protein hyperphosphorylation with site specificity. Studies have reported that repeated corticosterone injection in male Wistar rats results in no significant changes in Tau hyperphosphorylation at pSer199 and AT8 sites in hippocampal neurons, but increased expression at pSer396 (45). Tau hyperphosphorylation at pSer404 was induced in primary cell cultures of cortical neurons in mice by adding a beta peptide into the culture medium; the Tau hyperphosphorylation was not found at Thr205 (46). Research has shown if Tau protein is hyperphosphorylated at pSer396 and pSer404, Tau hyperphosphorylation is induced at Ser199 when plasmids are transferred into human embryonic kidney cells (47). The hyperphosphorylation of Tau protein at pSer396 in AD patients contributes to its dissociation from microtubules and affects the stability of microtubules (48). Hyperphosphorylation of Tau protein at pSer396 and pSer404 leads to a reduction in its solubility and the formation of NFTs (49).

Tau protein hyperphosphorylation at Ser396/404 and Ser422 in AD patients can also enhance the mutual polymerization of Tau protein to form NFT, while hyperphosphorylation at Ser262 can weaken its polymerization ability (50–52). We found that the phosphorylation sites of Tau protein are different in different diseases and animal studies. Therefore, we speculated that phosphorylation sites of pSer396 and pSer404 may be more sensitive to CUMS in the pathogenesis of depression. In our next experiment, we will closely study different phosphorylation sites of Tau protein that may be related to the development of depression. We will try to observe whether the Tau hyperphosphorylation sites in different models are consistent with the Tau hyperphosphorylation sites induced by CUMS and detect the sites of Tau hyperphosphorylation in cerebrospinal fluid of patients who have clinical depression with different degrees of severity. In addition, the specific mechanism by which phosphorylated Tau protein aggregates in hippocampal synapses to cause a decrease in GluR1 should be studied further.

## Ethics Statement

This study was carried out in accordance with the recommendations of the “Institutional Animal Care and Use Committee of China Medical University.” The protocol was approved by the “Institutional Animal Care and Use Committee of China Medical University.”

## Author Contributions

XWu and GW conceived and designed the experiments. YLi, RD, XR, WR, HYa, and YT performed the experiments. HYu, XZ, JY and XWa helped to analyze and interpret the data. GW drafted the manuscript. XWu, EX, YLu, and GZ provided critical revisions. All the authors reviewed and approved the final manuscript.

## Funding

The present study was supported by grants from the National Natural Science Fund of China (81171032, 81100807, and 81671867), the Natural Science Foundation of Liaoning, China (2015020514), and the Research Project of Liaoning Department of Education (L2014316).

## Conflict of Interest Statement

The authors declare that the research was conducted in the absence of any commercial or financial relationships that could be construed as a potential conflict of interest.

The handling editor declared a shared affiliation, though no other collaboration, with several of the authors GW, HY, RD, XR, YT, WR, HY, XZ, XW, EX, JY, GZ, YL, and XW at the time of the review.

## Abbreviations

AD, Alzheimer’s disease; AMPA, α-amino-3-hydroxy-5-methyl- 4-isoxazolepropionic acid; ANOVA, one-way analysis of variance; BDNF, brain-derived neurotrophic factor; CUMS, chronic unpredictable mild stress; eEF2, eukaryotic elongation factor 2; EPM, elevated plus-maze; FST, forced-swim test; GluA1, glutamate receptor 1; KO, knockout; LTD, long-term depression; LTP, long-term potentiation; m-TOR, rapamycin target protein; NFT, neurofibrillary tangles; NMDA, N-methyl-D-aspartate; OFT, open-field test; PSD95, postsynaptic density protein 95; PVDF, polyvinylidence difluoride; SDS-PAGE, sodium dodecyl sulfate polyacrylamide gel electrophoresis; SPT, sucrose-preference test; TBST, Tris-buffered saline–Tween.
